# Supplementary material for: Water-Soluble Chalcogenide W6-Clusters: On the Way to Biomedical Applications
Source: Int J Mol Sci. 2022 Aug 5;23(15):8734. doi: 10.3390/ijms23158734 (PMC9369320; doi:10.3390/ijms23158734)
Supplement: Supplementary file 1 [file ijms-23-08734-s001.zip › ijms-1848282-supplementary.pdf]

Water-Soluble Chalcogenide W_6-Clusters: On the Way to Biomedical Applications

Alena D. Gassan [1,2], Anton A. Ivanov [1], Tatiana N. Pozmogova [1,2], Ilia V. Eltsov [2], Natalia V. Kuratieva [1], Yuri V. Mironov [1,*] and Michael A. Shestopalov [1]

1 Nikolaev Institute of Inorganic Chemistry of Siberian Branch of Russian Academy of Sciences, 3 Acad. Lavrentiev ave., 630090 Novosibirsk, Russia
2 Department of Natural Sciences, Novosibirsk State University, 1 Pirogova st., 630090 Novosibirsk, Russia
* Correspondence: yuri@niic.nsc.ru

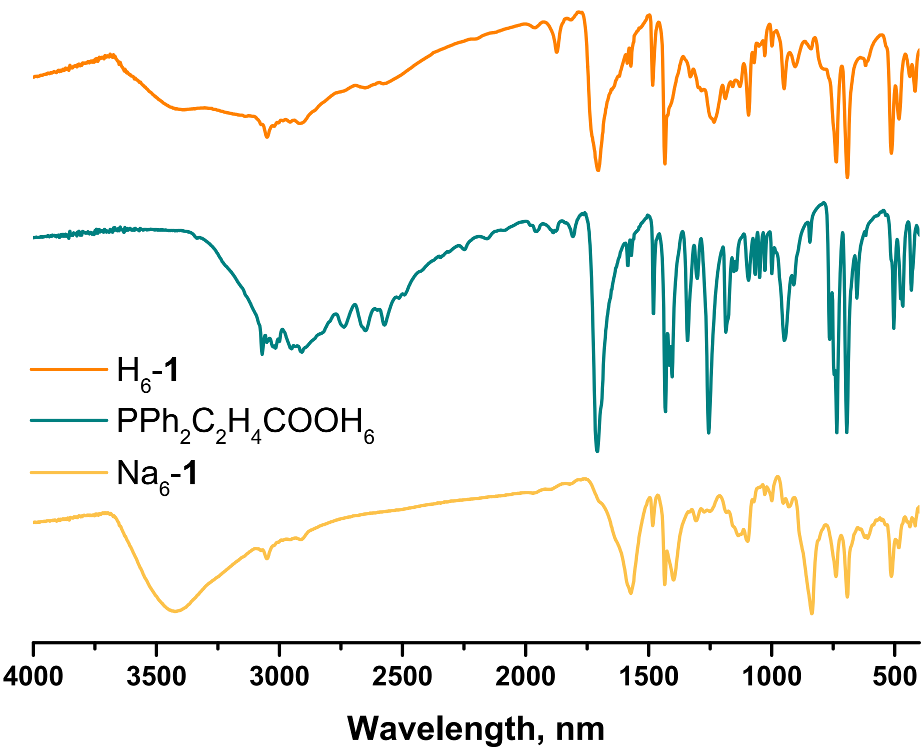

Figure S1. FTIR spectra of H_6-**1**, Na_6-**1**, in comparison with $PPh_2C_2H_4COOH$.

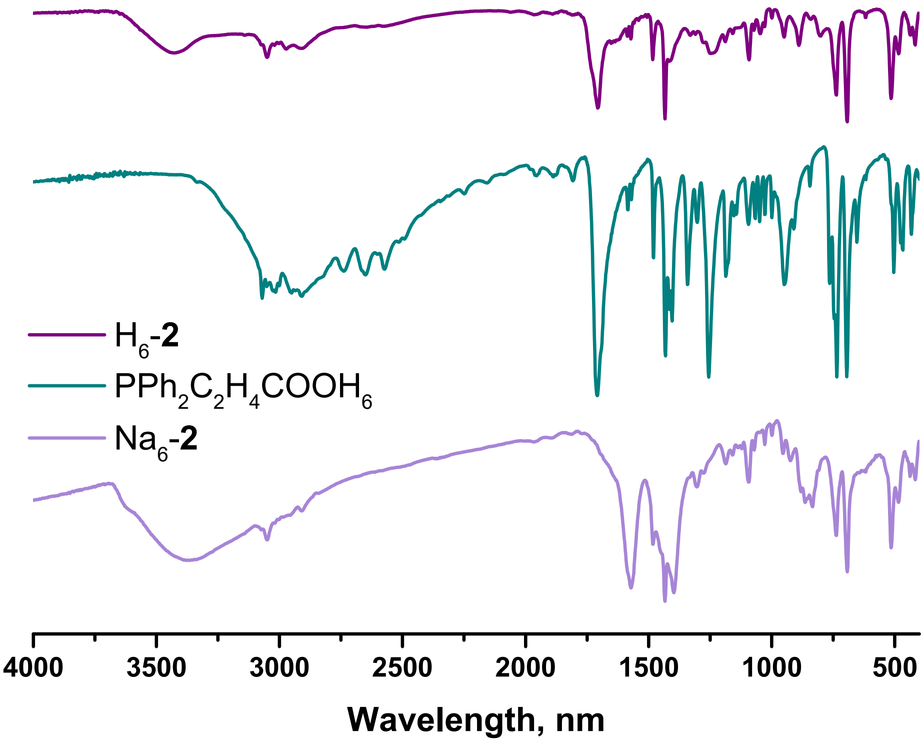

Figure S2. FTIR spectra of H_6-**2**, Na_6-**2** in comparison with $PPh_2C_2H_4COOH$.

Table S1. Selected crystallographic parameters of the single-crystal X-ray diffraction structural analysis for H_6-**1**·7H_2O·2.5Et_2O, H_6-**2**·3H_2O·Et_2O and Na_6-**1**·7.5H_2O·Me_2CO

Compound	H_6-**1**·7H_2O·2.5Et_2O	H_6-**2**·3H_2O·Et_2O	Na_6-**1**·7.5H_2O·Me_2CO
Empirical formula	$C_{98}H_{124}O_{21.5}P_6S_8W_6$	$C_{94}H_{100}O_{16}P_6Se_8W_6$	$C_{93}H_{105}Na_6O_{20.5}P_6S_8W_6$
Formula weight	3191.36	3406.33	3234.10
Temperature, K	150(2)	150(2)	150(2)
Crystal system	triclinic	triclinic	triclinic
Space group	$P\bar{1}$	$P\bar{1}$	$P\bar{1}$
a, Å	14.2426(2)	14.3920(4)	14.1628(11)
b, Å	15.2223(3)	15.2716(4)	15.2892(11)
c, Å	17.0620(3)	17.2025(5)	17.1646(13)
α, °	98.507(1)	96.687(1)	76.380(2)
β, °	108.605(1)	110.668(1)	72.995(2)
γ, °	114.885(1)	115.038(1)	71.046(2)
V, $Å^3$	3004.13(9)	3043.49(15)	3320.8(4)
Z	1	1	1
ρ_{calc}, g/cm^3	1.764	1.859	1.617
μ, mm^{-1}	5.999	8.168	5.445
F (000)	1546	1598	1555
Crystal size	0.17 × 0.07 × 0.04	0.25 × 0.09 × 0.05	0.30 × 0.16 × 0.01
2Θ range for data collection, °	2.363 – 27.572	2.304 – 27.234	1.761 – 26.692
Index ranges	$-18 \le h \le 18$<br>$-19 \le k \le 19$<br>$-22 \le l \le 21$	$-18 \le h \le 18$<br>$-19 \le k \le 19$<br>$-22 \le l \le 21$	$-17 \le h \le 17$<br>$-19 \le k \le 19$<br>$-18 \le l \le 21$
Reflections collected	28904	27490	27675
Independent reflections; [R_{int}]	13774; [0.0441]	13480; [0.0357]	13729; [0.0459]
Parameters refined	701	641	639
Goodness-of-fit on F^2	1.031	1.049	1.094
R_1 / wR_2(I > 2σ(I))	0.0417 / 0.0920	0.0392 / 0.1125	0.0618 / 0.1718
R_1 / wR_1 (all data)	0.0622 / 0.0975	0.0653 / 0.1218	0.1004 / 0.1877
$\Delta\rho_{max}/\Delta\rho_{min}$ (e·$Å^{-3}$)	1.583 / –1.176	2.618 / –1.171	6.488 / –2.466

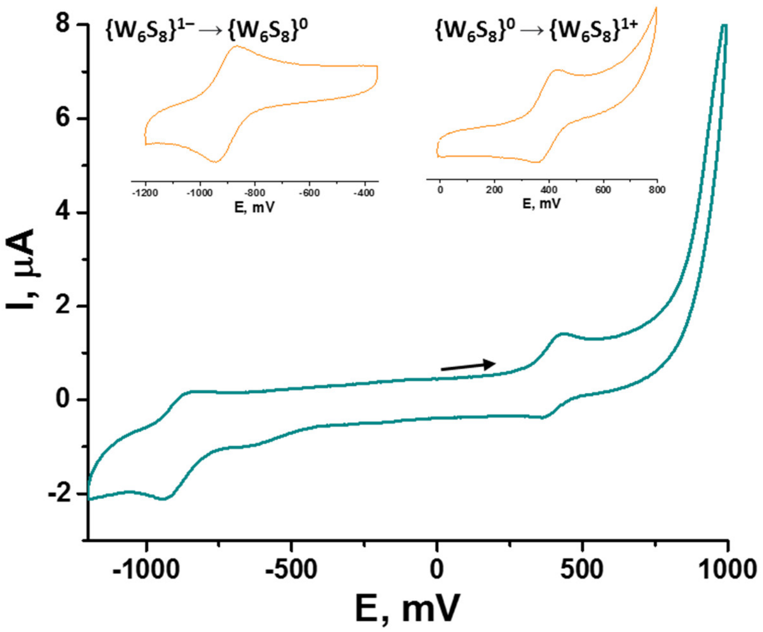

Figure S3. Cyclic voltammetry of the H$_6$-**1** (0.5 mM) in 0.1 M Bu_4NClO_4 DMSO solution, scan rate – 100 mV/s. Reference electrode – Ag/AgCl/3.5 M KCl.

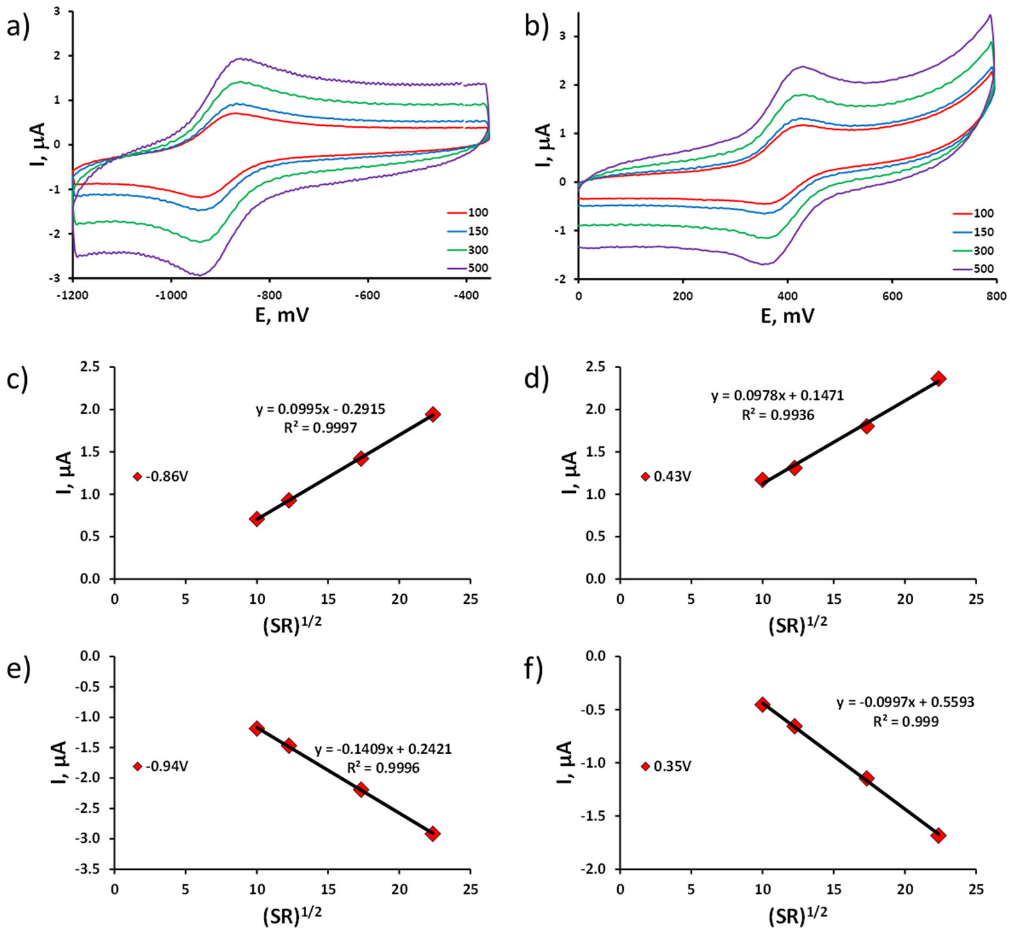

Figure S4. Cyclic voltammograms of H$_6$-**1** at various scan rate (**a**,**b**) and linear dependence of the cathodic (**c**,**d**) and anodic (**e**,**f**) peak current upon square root of the potential scan rate.

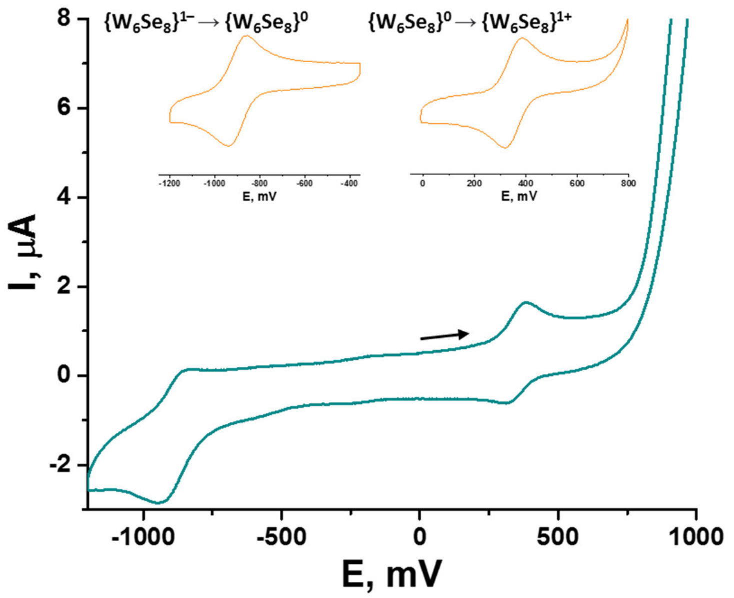

Figure S5. Cyclic voltammetry of the H_6-**2** (0.5 mM) in 0.1 M Bu_4NClO_4 DMSO solution, scan rate – 100 mV/s. Reference electrode – Ag/AgCl/3.5 M KCl.

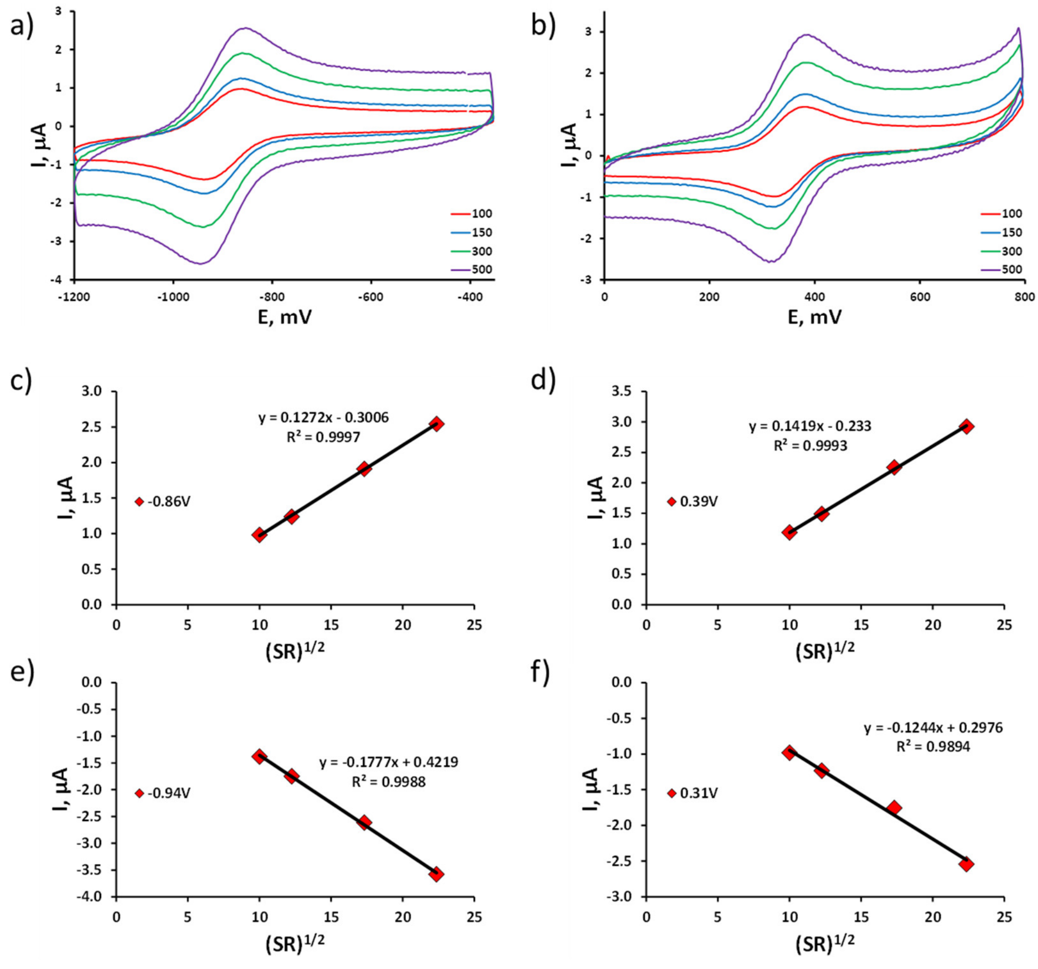

Figure S6. Cyclic voltammograms of H_6-**2** at various scan rate (**a**,**b**) and linear dependence of the cathodic (**c**,**d**) and anodic (**e**,**f**) peak current upon square root of the potential scan rate.

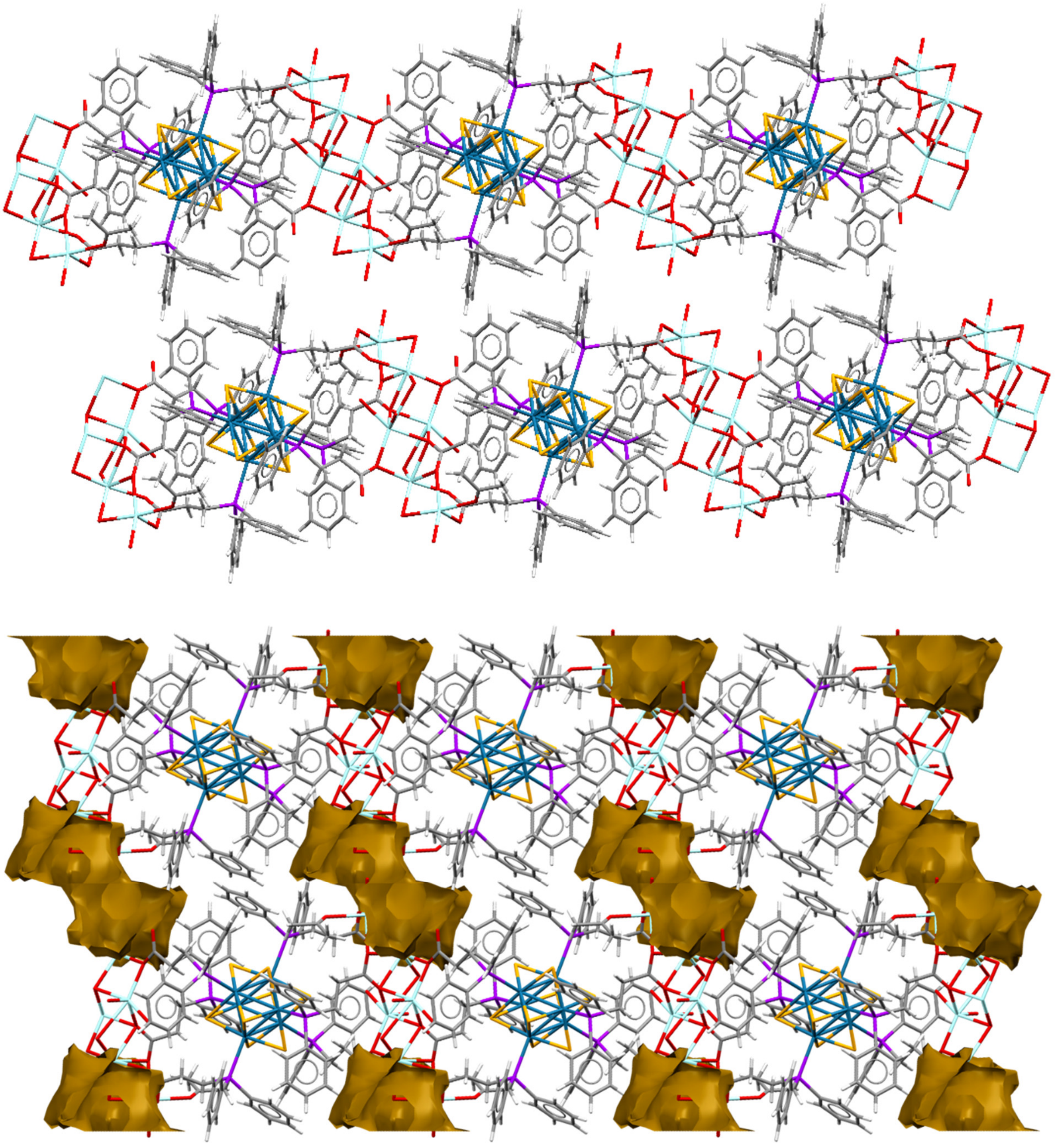

Figure S7. Chains -{alkali cations-cluster anions}- in Na_6-**1** crystal structure (top) and shape of the solvent accessible surface of the voids, illustrated along the *a* axe, calculated by the Mercury CSD 2021.3.0 program with default parameters (1.2 Å probe radius, 0.7 Å approximate grind spacing) (bottom).

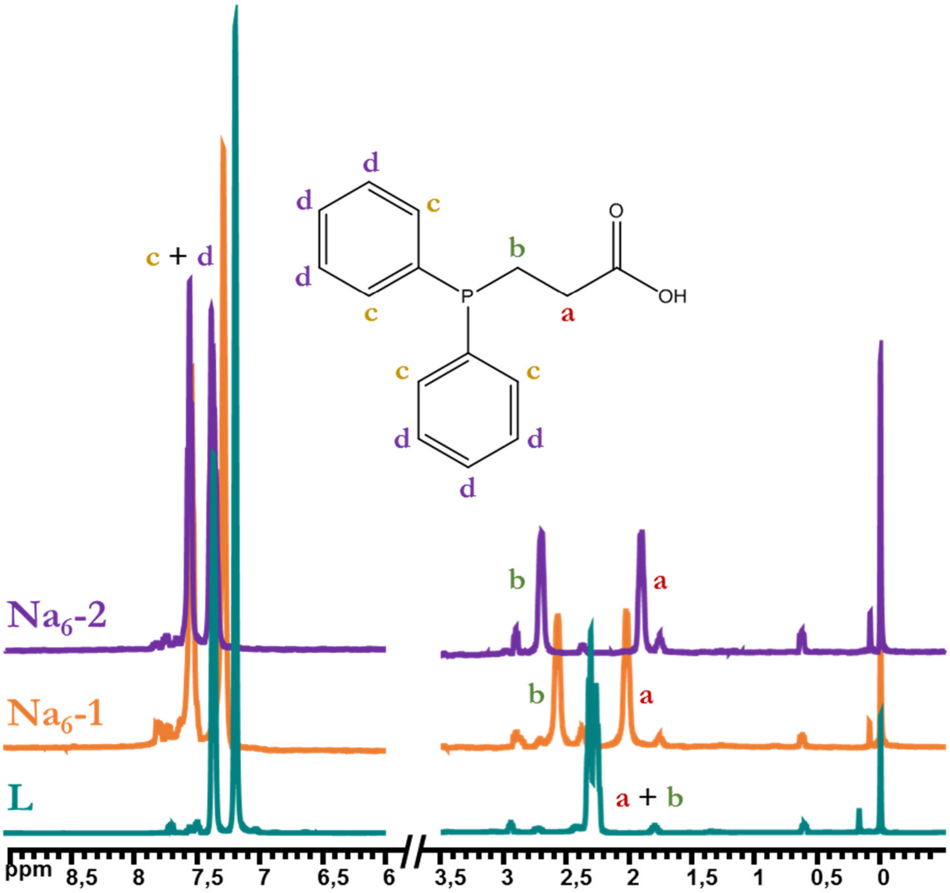

Figure S8. ^{1}H NMR spectra of Na$_6$-**1** and Na$_6$-**2** in D_2O in comparison with $PPh_2C_2H_4COOH$.

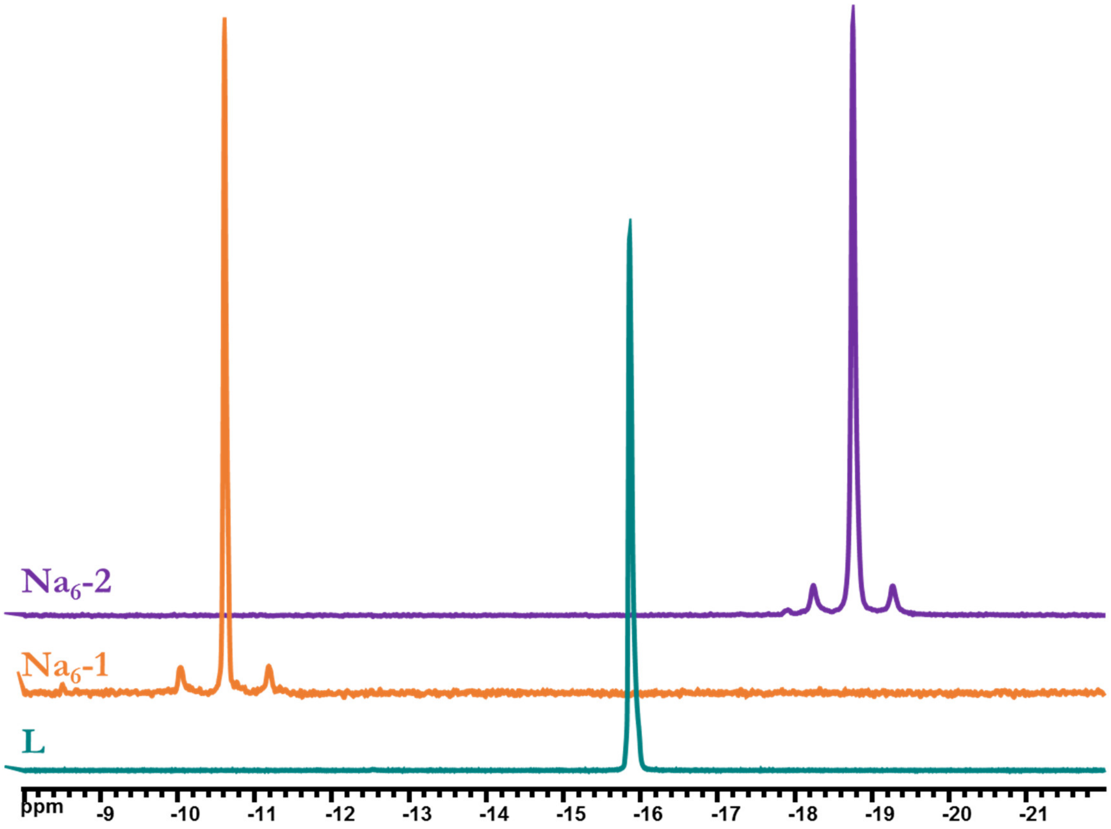

Figure S9. ^{31}P NMR spectra of Na$_6$-**1** and Na$_6$-**2** in D_2O in comparison with $PPh_2C_2H_4COOH$.

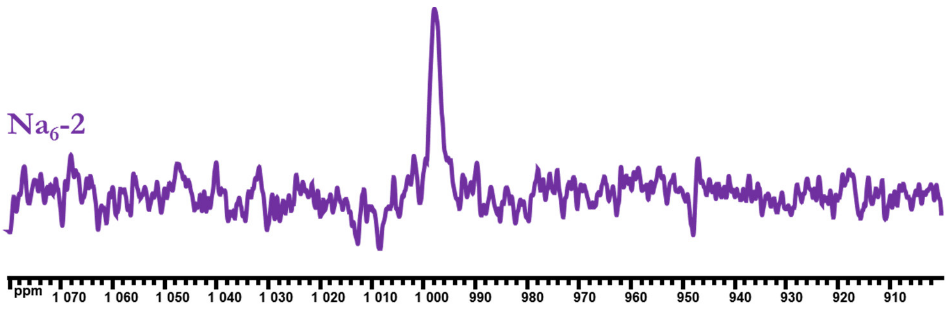

Figure S10. ^{77}Se NMR spectra Na_6-**2** in D_2O.

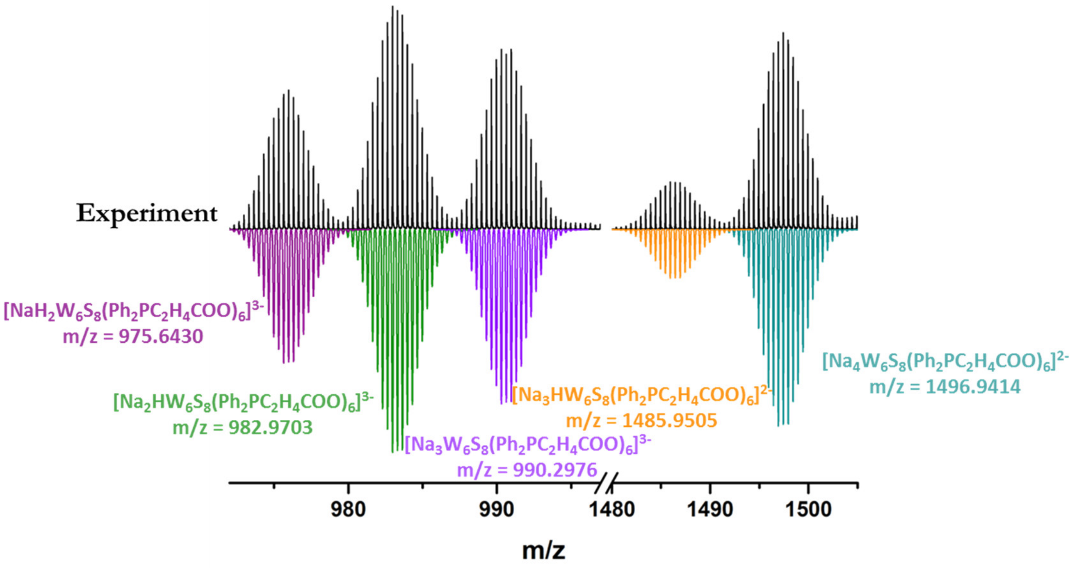

Figure S11. Fragments of mass-spectrum of Na_6-**1** in aqueous solution (black) and simulated profiles of forms (colored).

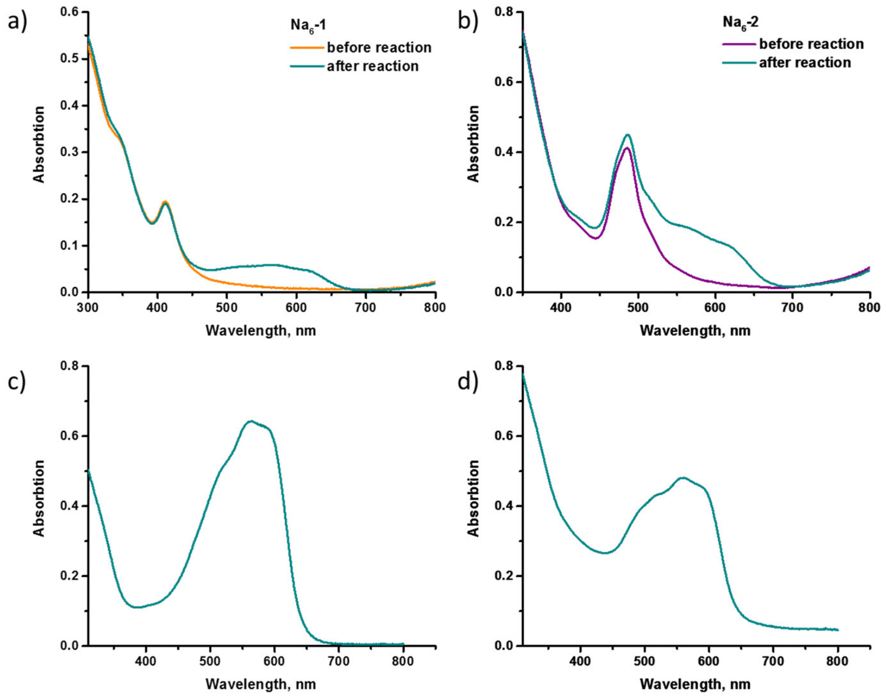

Figure S12. UV/Vis spectra of aqueous solutions of Na_6-**1** (a) and Na_6-**2** (b) after and before reactions with MTT and of violet precipitate after reaction with Na_6-**1** (c) and Na_6-**2** (d) in ^{i}PrOH.

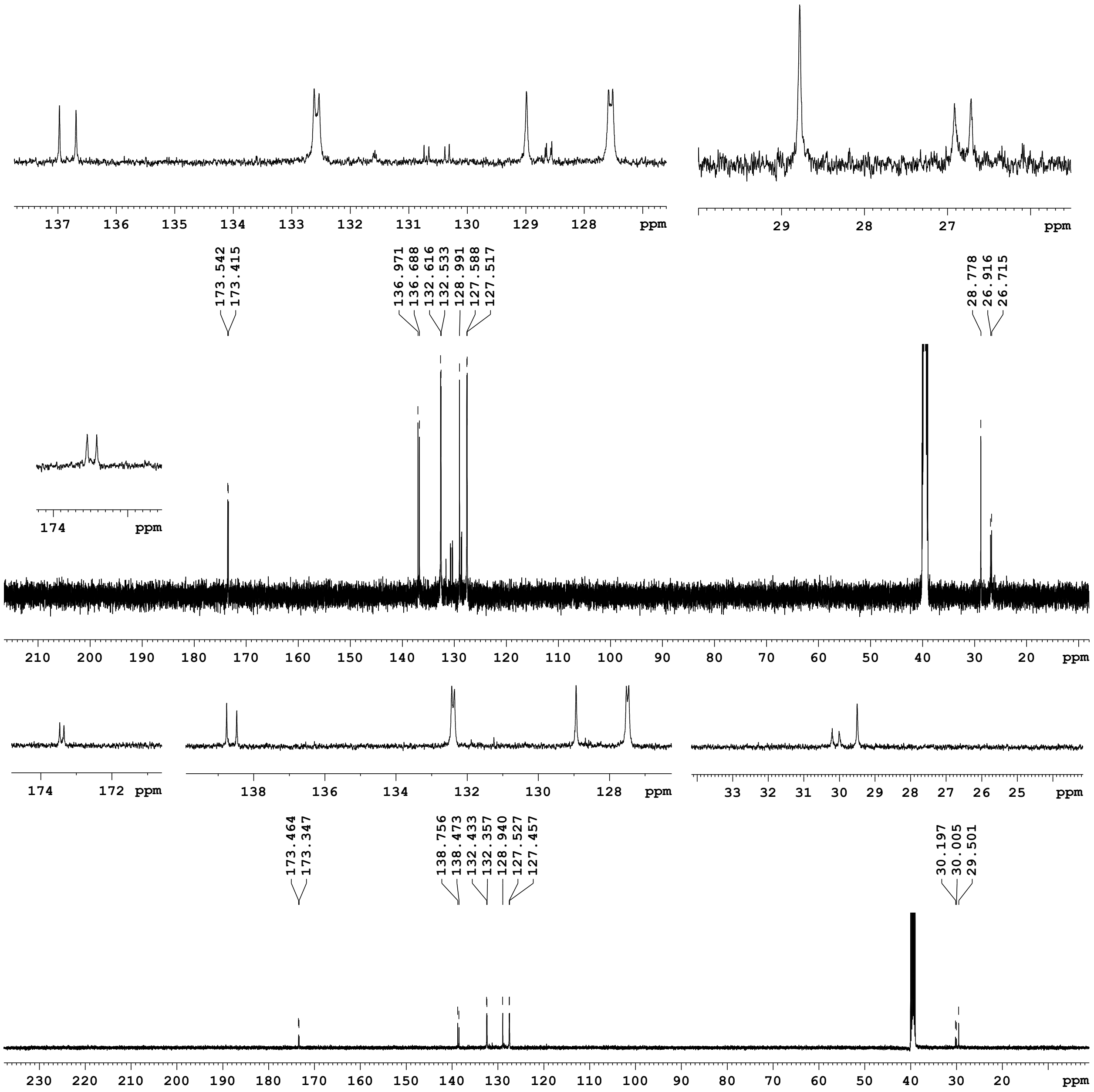

Figure S13. ^{13}C NMR spectrum for H_6-**1** (top) and H_6-**2** (bottom) in DMSO-d_6.

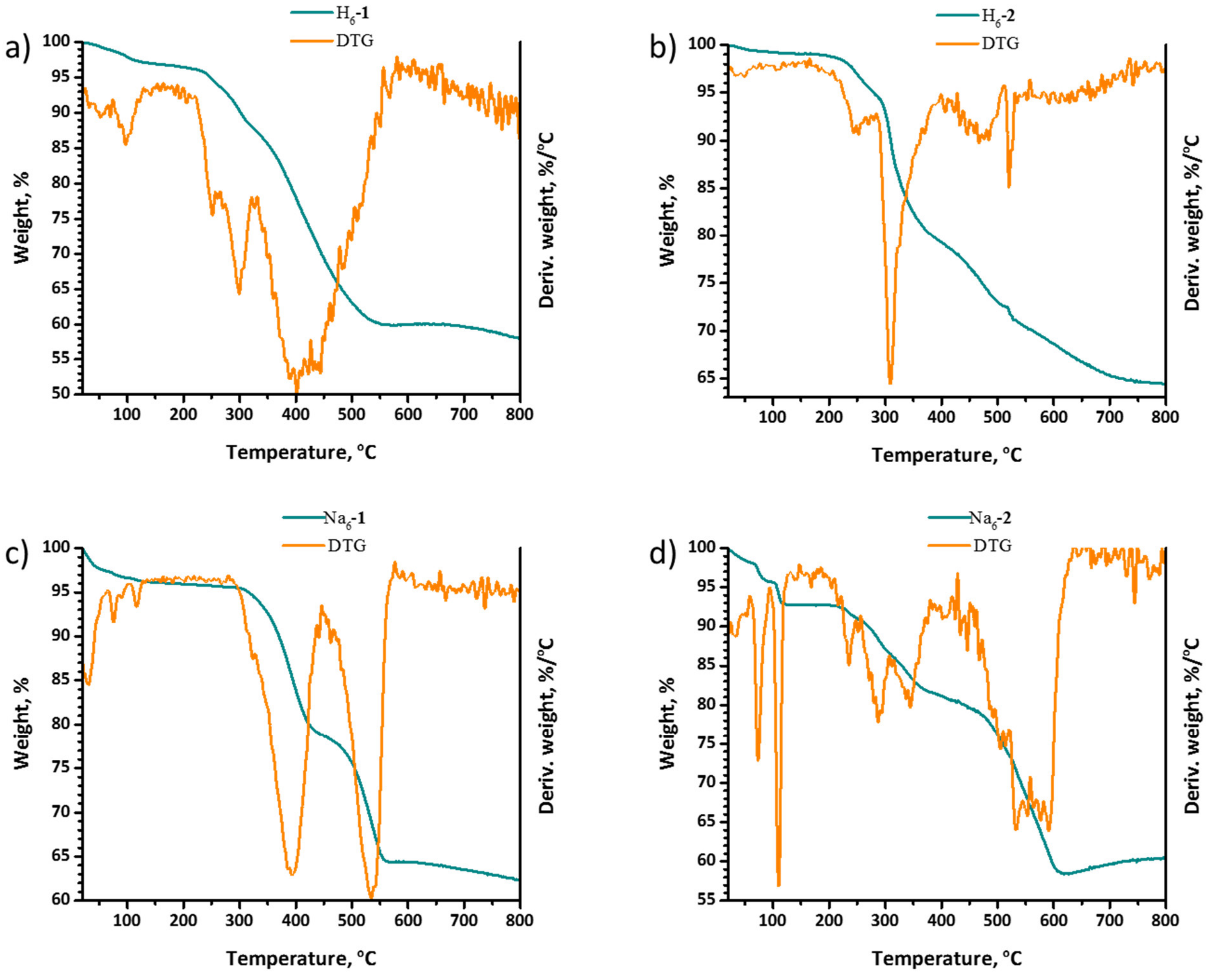

Figure S14. TGA and DTG curves for H_6-**1** (a), H_6-**2** (b), Na_6-**1** (c) and Na_6-**2** (d). Heating rate of 10°C·min^{-1}.

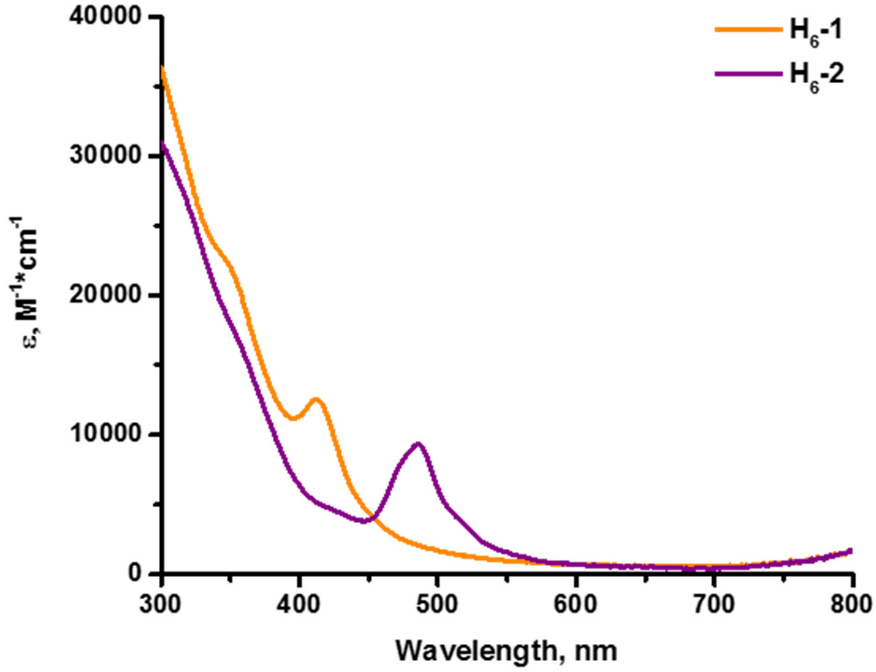

Figure S15. UV/Vis spectra of H_6-**1** and H_6-**2** in DMSO.

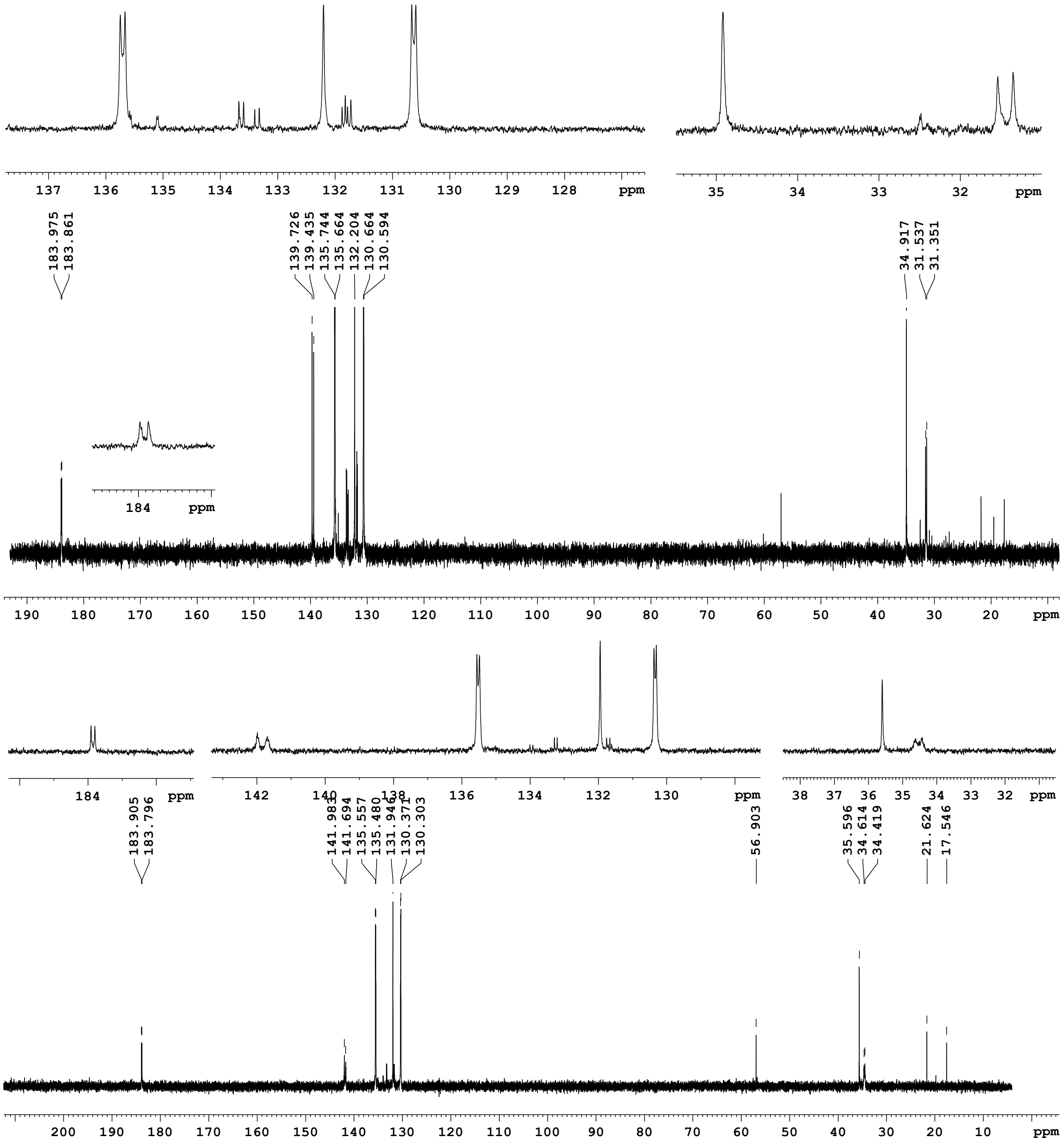

Figure S16. ^{13}C NMR spectrum for Na_6-**1** (top) and Na_6-**2** (bottom) in D_2O.
